# Plant DNA Barcode as a Tool for Root Identification in Hypogea: The Case of the Etruscan Tombs of Tarquinia (Central Italy)

**DOI:** 10.3390/plants10061138

**Published:** 2021-06-03

**Authors:** Daniela Isola, Flavia Bartoli, Simone Langone, Simona Ceschin, Laura Zucconi, Giulia Caneva

**Affiliations:** 1 Department of Sciences, Roma Tre University, 00146 Rome, Italy; flavia.bartoli@uniroma3.it (F.B.); simone.langone@gmail.com (S.L.); simona.ceschin@uniroma3.it (S.C.); giulia.caneva@uniroma3.it (G.C.); 2 Department of Ecological and Biological Sciences (DEB), University of Tuscia, 01100 Viterbo, Italy; laura.zucconi@unitus.it

**Keywords:** archaeological sites conservation, biodeterioration, herbaceous roots damages, Monterozzi Necropolis, mural paintings conservation, plant molecular markers, root damage management, root risk assessment, subterranean cultural heritage, vegetation management

## Abstract

Roots can produce mechanical and chemical alterations to building structures, especially in the case of underground historical artifacts. In archaeological sites, where vegetation plays the dual role of naturalistic relevance and potential threat, trees and bushes are under supervision. No customized measures can be taken against herbaceous plants lacking fast and reliable root identification methods that are useful to assess their dangerousness. In this study, we aimed to test the efficacy of DNA barcoding in identifying plant rootlets threatening the Etruscan tombs of the Necropolis of Tarquinia. As DNA barcode markers, we selected two sections of the genes *rbcL* and *matK*, the nuclear ribosomal internal transcribed spacer (nrITS), and the intergenic spacer *psbA-trnH*. All fourteen root samples were successfully sequenced and identified at species (92.9%) and genus level (7.01%) by GenBank matching and reference dataset implementation. Some eudicotyledons with taproots, such as *Echium italicum* L., *Foeniculum vulgare* Mill., and *Reseda lutea* L. subsp. *lutea*, showed a certain recurrence. Further investigations are needed to confirm this promising result, increasing the number of roots and enlarging the reference dataset with attention to meso-Mediterranean perennial herbaceous species. The finding of herbaceous plants roots at more than 3 m deep confirms their potential risk and underlines the importance of vegetation planning, monitoring, and management on archaeological sites.

## 1. Introduction

Vegetation plays a dual role on archaeological sites. Plants contribute significantly to the characterization of landscapes, enhancing their naturalistic, ecological, and cultural value [1,2,3,4,5,6,7,8]. However, vascular plants, especially trees, can seriously threaten the conservation of ancient monuments as they can directly colonize walls and damage structures by root expansion [1,9,10,11,12,13,14,15]. This risk can be highly relevant in the case of underground ruins as there might be a short distance between the buried archaeological structures and the vegetated ground level. Damages caused by roots have been reported for hypogeal tombs, including the Christian and Jewish catacombs [16], Mithraea, temples, and underground villas such as the Domus Aurea [17,18] and the Domus Tiberiana in Rome [19]. Roots with their growth may produce mechanical and chemical damages on foundations, mortars, plasters, walls, and frescoes [15,16,20,21], even dislodging large stones and weakening the mineral wall matrix and masonry texture by the release of chemical compounds [15]. Moreover, roots can be considered particularly detrimental in hypogea since they may favor water penetration (Figure 3E,F) and affect the internal microbial community [22,23]. Several studies evidenced that roots can modify the diversity and richness of the resident community into this fragile oligotrophic environment. Roots carrying exogenous rhizosphere microorganisms and organic carbon sources (as root litter and exudates) [24,25,26] can favor the growth and spread of detrimental heterotrophs.

The control of vegetation on archaeological sites has been addressed by several authors since the 1980s [1,11,17,27,28]. Particular attention has been paid to the classification of risk assessment tied to individual plant species and plant communities starting from species identification and information on relevant plant elements such as life form (according to Raunkiær), invasiveness, size, shape, and vigor of roots [29]. In this light, a hazard index (HI) ranging from 0 to 10 is assigned to each species. Recently, the ecological characteristics of the different plants in response to diverse micro-environmental (i.e., exposure and inclination) and micro-edaphic conditions (i.e., soil availability and composition) have been considered as additional parameters in the risk assessment [8,12].

Plant identification represents the basic step for the risk assessment, aimed to design a vegetation control plan with periodic monitoring and checks for undesired growth on archaeological sites [9]. Trees and bushes are given special attention on these sites, and their lignified roots can be identified through morpho-anatomical characters supported by the comparison with the nearby aboveground plant species [16,17]. However, morpho-anatomical identification is sometimes time-consuming and may be difficult when immature or ruined specimens lack one or more fundamental characters for their taxonomical identification [30]. In the case of primary roots, the identification is much more complex due to the substantial similarities of the stele organization among the different species. Moreover, the root system is affected by phenotypic plasticity so plants with identical genotypes adapt and modify their root system architectures based on the biotic and abiotic environmental factors [31]. Concerning the root penetration of herbaceous species and their architecture, very few data exist, limited to some important reviews [32,33]. Attempts to identify primary roots have been performed for the Etruscan tombs in Latium and Tuscany, where root penetration represents an enduring problem [2,23,34,35,36,37]. For example, cultures of root meristems, coupled with the analysis of the aboveground vegetation, were performed, but the limitations and difficulties of such methods were stressed [37].

In the last 15 years, DNA barcoding has become a primary tool for fast species identification. DNA sequence data from standard genome regions are routinely used in several applications: biomonitoring, invasive species identification, food fraud, forensics, etc. [38,39,40,41,42]. Although the nuclear Internal Transcribed Spacer (ITS) and the mitochondrial *cox1* gene (Cytochrome c oxidase I) are universally used for fungi and animals, respectively, there is no strong consensus on which DNA regions should be used for plants (Fourth International Barcode of Life Conference, www.dnabarcodes2011.org, accessed on 6 January 2021). Two plastid coding regions, *rbcL* and *matK*, were suggested as a core-barcode for plants by the Plant Working Group of the Consortium for the Barcode of Life [43,44,45]. However, due to their differential discriminatory power across taxa, additional regions were also recommended, such as the plastid intergenic spacer *psbA-trnH* and the rapidly evolving Internal Transcribed Spacers (ITS) of nuclear ribosomal DNA [43,44,45,46,47,48,49]. As such, it is useful for a closer evaluation of the power and possible limits of this method.

We aimed to evaluate the power of the DNA barcode method in identifying higher plants starting from herbaceous roots and to test its application in the cultural heritage field, for the first time. The use of this method potentially has a great relevance when roots occur in underground layers and only roots are available for the identification of the plant species (i.e., when the aerial parts of a plant are not developed or visible). In the frame of an international cooperation project focused on biodeterioration and conservation of underground monuments, we analyzed the Necropolis of Monterozzi in Tarquinia (central Italy), where many painted hypogeal tombs are threatened from the penetration of rootlets and, consequently, are in need of conservation actions.

## 2. Results

### 2.1. DNA Marker Performances and Root Identification

**Figure 1:**
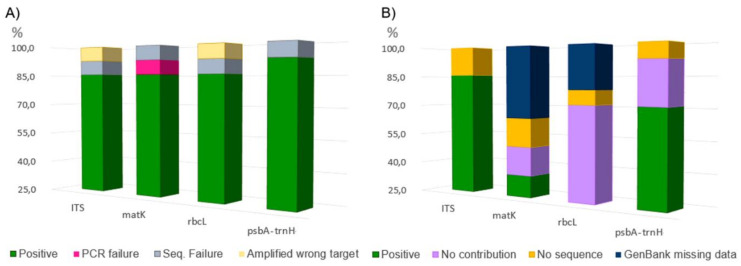
Molecular marker performances (%). Sequencing performances, namely the frequencies on which were obtained high quality sequences (green) and technical affecting factors (**A**). Contribution to species identification, namely the frequencies on which Blast comparison returned a single reliable best match (green) and affecting factors (**B**). No statistical analysis was performed due to the number of samples.

**Figure 2:**
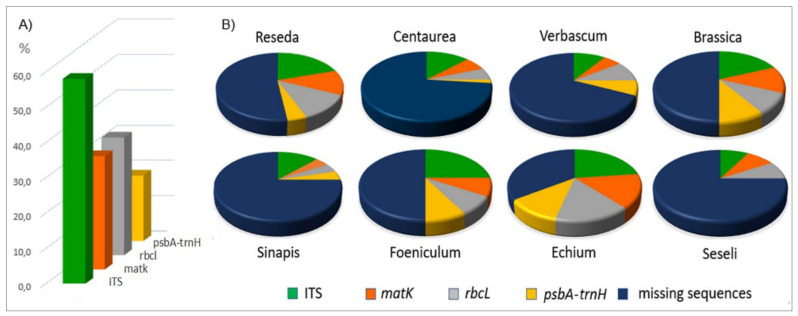
GenBank sequence coverage of the eight plant genera identified by root sequencing, showing the coverage percentages by molecular marker (**A**) and genus (**B**).

**Figure 3:**
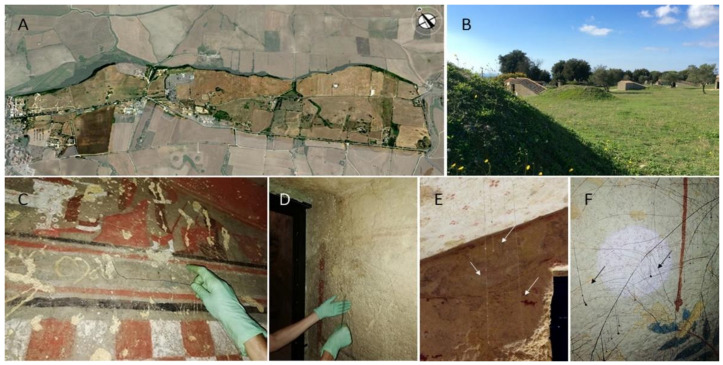
Study area and root sampling: aerial view of the Monterozzi necropolis at Tarquinia (**A**); the landscape with tumuli (**B**); root sampling (**C**,**D**); and water droplets along roots, as highlighted by arrows (**E**,**F**).

We obtained readable sequences from all 14 root extracts (Table S1), belonging to eight genera. Overall, 78.57% of the samples were successfully sequenced for all the chosen molecular markers (Figure 3A). The matK target gene was more difficult to be amplified than others, as it was frequently necessary to repeat the amplifications using different primer sets. Nevertheless, 7.14% *matK* PCR resulted as negative. The highest incidence of successful sequencing was recorded with the *psbA-trnH* target (13 out of 14). As for drawbacks, using primers for ITS and *rbcL* regions (one time each), portions of fungal and mitochondrial genome were amplified (Figure 1 and Table 1). Figure 2B shows that technical factors, namely GenBank missing data and failure in PCR/sequencing, affected the contribution of each marker gene in the sample identification. Marker features such as the ability, or not, to return a single best match were also considered (recorded as “no contribution”, Figure 2B). In this light, it is possible to note each marker contribution in root identification at the species level. ITS was useful for the species identification in 85.71% of the cases, followed by *psbA-trnH* and *matK* with 64.29% and 35.71%, respectively. The *rbcL* sequences, not returning a sole best match, were not useful for species identification in 71.43% of the cases, while *matK* always returned a single species as the best match, even if this was not resolutive for Samples C1 and M1 (Table 1). A perfect match (100%) was recorded for *Reseda crystallina* Webb & Berthel. and an almost perfect match (99.83%) for *Reseda lutea* L.; however, as this match was in contrast with the results achieved by ITS and *psbA-trnH* sequences (Table 1), it was considered not reliable.

As reported in Table 1, GenBank BLAST comparison was fruitful in identifying *Foeniculum vulgare* Mill. (21.4% of samples; Samples H2, CP01, and CP02), *Echium italicum* L. (14.3% of roots processed; Samples M2 and 02), *Sinapis alba* L. (Sample D4), and *Reseda lutea* (14.3% of samples; Samples C1 and M1). Further investigations are needed for the identification of the remaining root samples belonging to the genera *Centaurea* (Samples C2, LT01, and LT3, possibly *Centaurea aspera* L.), *Brassica* (Sample F1), *Verbascum* (Samples F3), and *Seseli* (Sample 01). The recurrence of some species was also evidenced.

### 2.2. Integrated Taxonomic Identification Method

BLASTn best matches (Table 1) were merged with local flora data to remove the alien species. This was the case of *Reseda crystallina*, which was previously considered not reliable despite the perfect match obtained (100%, matk, Samples M1 and C1) and now definitively discarded because does not belong to the Italian flora. 

The genera of interest are not fully represented in the GenBank database (Figure 2 and Table S2). The highest number of sequences was recorded for the nuclear ITS, covering 58.02% of the local species, followed by *rbcL*, *matk*, and the intergenic *psbA-trnH* spacer with 33.33%, 32.10%, and 18.52%, respectively (Figure 2A). The poor representation of the local flora became more evident by genera. For example, 51.61% of *Centaurea* local species and subspecies are represented in GenBank with at least a single record each, and even lower is the occurrence for *Seseli* (33.33%) (Table S2). Local flora represented by at least two molecular markers per species ranges from 38.8% for *Verbascum* to 0% for *Seseli*. No sequences for the *psbA-trnH* intergenic spacer are present for *Seseli* (Figure 2B).

The six plants taken in the field, namely *Centaurea aspera* L. subsp. *aspera*, *Reseda lutea* L. subsp. *lutea*, *Seseli tortuosum* L. subsp. *tortuosum*, *Verbascum sinuatum* L., and two *Diplotaxis* (*D. erucoides* (L.) DC. and *D. tenuifolia* (L) DC.), were sequenced for all considered targets (except *V. sinuatum* ITS); all sequencing results and the relative GenBank accession number are shown in Table S3.

The *Diplotaxis* sp. were the only Brassicaceae species that we found along the visitors’ path. We also had root samples that showed matches with *Sinapis alba* and *Brassica* sp. The tombs where we initially collected the samples were later inaccessible (due to the COVID-19 restrictions). Due to the low number of samples processed starting from leaf extracts and the incomplete overlapping of the species considered, it was not possible to perform a statistical analysis to assess if the differences found in sequencing success yields (root vs. leaf sequencing) are significant.

The comparison of the root sequences with the new reference sequences allowed the identification of several species, e.g., *S. tortuosum* subsp. *tortuosum* (Tomb 5512), *V. sinuatum* (Tomb of the Sculptures), *R. lutea* subsp. *lutea* (the Moretti, M1, and Lotus flower, C1 tombs), and *C. aspera* subsp. *aspera* (Lotus Flower tomb, Samples C2, LT01, and LT3). Besides, no root matches were recorded with the species *D. tenuifolia* and *D. erucoides*, which commonly grow among the tombs in the aboveground area along the visitors’ path.

## 3. Discussion

There is a limited number of studies on roots’ identification by DNA barcode, mainly focused on the plant roots’ distribution and diversity in the belowground or aimed at authenticating medicinal plants [50,51,52,53,54]. On archeological sites, trees and bushes are commonly maintained under strict control, while no information and neither preventive measures nor guidelines are issued for herbaceous plants. This is mainly due to the general assumption that herbaceous plants are not dangerous, not deeply penetrating, and the lack of reliable and fast methods to identify plants starting from small, tiny roots. Indeed, the difficulty in identifying herbaceous roots allowed us to test the DNA barcoding efficiency.

Plant cells have three different genomes: nuclear, plastid, and mitochondrial [55]. Species, cell type, and age of the tissue affect the number of copies of the nuclear genome and the number of organelles, respectively [55]. Polysaccharides, polyphenolics, and secondary metabolites produced by plants could decrease the quality of their DNA extracts [56]. In this preliminary study, despite some difficulties, promising results were achieved with our protocol, leading to a successful four-marker sequencing in 11 out of 14 root samples (78.57%). Meanwhile, the negative outcomes can provide cues for improvement to be applied in the next step of this research.

Young, healthy, and tender tissues (better if from leaf meristems) are the ideal choice for good quality/quantity DNA extracts, due to the higher number of cells and the low deposition of starch and secondary metabolites [56]. Otherwise, in subterranean environments, a sufficient number of young fresh root samples is often not available, and this factor may affect the results.

Species discrimination with plant barcodes is typically lower than for animals and fungi, using cox1 and ITS barcodes, respectively [45]. This is in part due to the lower rate of nucleotide substitution in the plastid genome, but also tied, for example, to hybridization, polyploidy, and low levels of intraspecific gene flow for plastid markers [57].

It is well known that levels of species discrimination greatly vary among taxa, and several DNA barcoding studies on plants analyzed the discriminating power of molecular data within relatively homogeneous groups, such as families or genera [44,45]. Among plastid regions, *rbcL* is the best characterized gene because it is easily retrievable across terrestrial plants, suitable for high-quality bidirectional sequences, and easy to align [43,45]. Because of the best performing multi-locus combinations for species discrimination, *rbcL* was chosen as core-barcode with *matK* despite its modest discriminatory power [43]. In our study, *rbcL* sequences (500–650 bp) were never resolutive when used alone, but they were enough to identify the closest “species group” sharing the highest identity score. This information was useful in the field sampling to address the search for spontaneous species, which was not securely identifiable, allowing us to overcome the gap of missing sequences in GenBank. It was also highlighted that, when using *rbcL* primers, it is possible to amplify mitochondrial regions, a common event recorded in the Brassicaceae family [58].

Even though the *matK* gene showed high levels of discrimination power among angiosperm species [57,59], the main problem we had was due to the incomplete representation of the herbaceous local flora in GenBank. 

The ITS target was the most useful DNA marker due to the number of sequences deposited in GenBank, with about 58.02% of congeneric species (and subspecies), and its recognized discriminatory power. It is characterized by an easy amplification, but as drawback the ITS of possible fungal endophytes can be amplified as well (recorded here as sequencing failure). Although the intergenic spacer *psbA-trnH* is demonstrated to be easily amplified and sequenced and useful for species identification (64.29% of samples) [60,61], it is poorly represented in GenBank (18.52% of congeneric species of the Latium flora). Its high sequence length variability, ranging from 152 to 851 bp in eudicotyledons, from 151 to 905 bp in monocotyledons, and from 283 to 1006 bp in gymnosperms [60], was useful in the lab practice to distinguish among different specimens just after an electrophoretic run (e.g., *Foeniculum vulgare* ca. 350 bp and *Verbascum sinuatum* ca. 600 bp).

In the light of these results, the four-target sequencing was useful to increase the identification rate and obtain more reliable results looking for consistent identity scores along with markers. Being the match scores tied to the specific fragment amplified (even within the same gene) and its length, a single perfect match does not provide a reliable identification. This is, for instance, what happened with the *R. crystallina* sequence match. Despite the full identity found in *matK* gene with this species, this result conflicted with the results achieved with the two other markers. Moreover, this species does not belong to the Italian flora. This evidence highlighted the importance of having more than a single discriminating marker for identification as well as the relevant contribution of the local floristic data.

Database improvement was, instead, crucial to achieve the 92.85% of identification at species and subspecies level (at the genus level for the remaining 7.15%), confirming the importance of comparisons with the aboveground vegetation. The improvement of the existing sequencing data on the autochthonous flora could be very useful, if not mandatory, to implement protection strategies for archaeological sites and underground buildings in general. Moreover, the enlargement of the reference database is necessary to assess the best marker barcodes for a faster and reliable identification.

From a conservation viewpoint, our results prove the herbaceous plants, typical of arid calcareous grasslands, can be a potential threat for hypogeal environments, as their roots were found more than 3 m deep (e.g., Sample M2). Indeed, all tombs of this study area are cut into a very porous (30–43% of porosity) yellowish limestone [62]. Being quite brittle, this stone does not offer great resistance to root penetration. Moreover, the xeric conditions that occur in summer in this Mediterranean site, may drive roots in search for water until they reach burial chambers, where the relative humidity is frequently between 90% and 100% [23]. The relevant deep growth is probably linked also to the fact that the most recurrent species, namely *C. aspera, E. italicum*, *F. vulgare*, *R. lutea*, *S. tortuosum*, and *V. sinuatum*, are biennial or perennial hemicryptophytes characterized by a vigorous root system. Other herbaceous annual species, such as *Brassica* sp. and *S. alba*, showed a well-developed root system as well. The recorded taxa belonged to the families Apiaceae, Asteraceae, Boraginaceae, Resedaceae, and Brassicaceae (eudicotyledons). Their vegetative growth varies [63], ranging from the medium *C. aspera* (30–60 cm high), *R. alba* (10–80 cm), *S. tortuosum* (20–70 cm), and *S. alba* (30–70 cm) to the medium-high *E. italicum* (35–100 cm) and *F. vulgare* (40–150 cm). Scant information is available about the architecture of their root system and behavior in drought conditions. A character shared by most of these species is the presence of taproots, probably able to penetrate more deeply than the adventitious roots of monocotyledons [32,33]. As roots were sampled at different depths and sites within the tombs, an accurate mapping of roots protrusion in different hypogea could provide useful information for conservation practices.

From an applicative side, there are several reasons to avoid large-scale interventions. It is well known that the vegetation generally benefits from policies designed to protect the archaeological site [64,65]. Moreover, the protection of the cultural heritage does not imply extensive and aggressive management routines (e.g., massive use of herbicide) especially when, as in our case, the archaeological area is also a site of naturalistic relevance. Vegetation affects the microclimate conditions of the sites and underground structures, decreasing the temperatures and increasing the humidity values. Recent studies in these Etruscan tombs showed some positive potential effects of plant cover in the stabilization of the local microclimate [66]. The negative counterpart is the role played by roots as carriers for rhizosphere microorganisms, water penetration, and organic carbon supply [22,24,26]. As previously stressed, being hypogea oligotrophic environments, these inputs could lead to a disequilibrium in the resident microbial communities and the spreading of further deteriogenous species [23,26]. Interestingly, the fungal strains sequenced by chance (Sample F1) showed the highest identity score with strains CCFEE 6623 and 6662 isolated previously from the Moretti tomb, deeply threatened by fungi [26].

## 4. Materials and Methods

### 4.1. Study Area

Due to its artistic and historic relevance, the Etruscan necropolis of Monterozzi in Tarquinia (Latium, Central Italy) (Figure 1A) has been included, together with those of Cerveteri, in the UNESCO World Heritage Site list since 2004. The tombs, dating from the 7th to the 3rd century BC, were dug in calcarenites banks (Macco stone) and lie at depths ranging from 2 to 8 m [23]. As with other hypogea, these tombs are characterized by a high humidity level, a stable temperature throughout the year, and limited air circulation [26,67]. The necropolis landscape (Figure 1B) is characterized by the presence of many tumuli (which gave the name to the area of Monterozzi). Most of them have been flattened by agricultural practices and others have been dismantled and partially rebuilt to protect the main chamber without considering the original shape [36,68].

The area falls within the Mediterranean macro-bioclimate, with a lower meso-Mediterranean thermotype and a lower subhumid ombrotype [23]. The presence of trees and shrubs is quite limited within the Monterozzi necropolis (Figure 1A,B), which is characterized mainly by ruderal synanthropic herbaceous vegetation, with annual and perennial herbaceous species typical of Mediterranean meadows. Despite the long anthropization and excavation history (since the 19th century), this area maintains a good level of naturalness, also linked to the low incidence of non-native species, and it is included among the protected area of SCI/SAC (IT6010028) of the European Directive 92/43/CEE “Habitat” for its naturalistic relevance. As commonly occurs in archaeological areas, the herbaceous vegetation is subject to periodic mowing.

### 4.2. Root Sampling, DNA Extraction, Amplification, and Sequence Comparison

Fourteen root samples arising from seven hypogeal tombs (Table S4) were aseptically collected between February and November 2019 (Figure 3C,D), placed in sterile bags, and stored at −20 °C until use. DNA was extracted from fresh root material (70–100 mg) using the Nucleospin Plant kit (Macherey-Nagel, Düren, Germany) following the manufacturer instructions. PCR reactions were performed using the BioMix (BioLine, Luckenwalde, Germany). The reaction solution was prepared with 12 μL of Biomix, 5 pmol of each primer, and about 30 ng of template DNA in a total volume of 25 μL. DNA barcoding analysis was performed using four different DNA markers: the plastid coding *rbcL* and *matK* genes and the noncoding *psbA-trnH* regions and the nuclear ITS. The different primer sets used and the annealing temperatures are listed in Table 2. Amplifications were carried out using the MyCycler™ Thermal Cycler (Bio-Rad Laboratories, Munich, Germany) applying the following protocol for plastid markers: an initial denaturation step for 2 min at 95 °C, 45 cycles at 95 °C for 30 s, annealing at 50 °C (or 53 °C as in Table 1) for 1 min 30 s, extension at 72 °C for 40 s, followed by a final extension at 72 °C for 5 min [52]. For ITS, PCR conditions were: initial denaturation for 3 min at 95 °C, 35 cycles of denaturation at 95 °C for 30 s, annealing at 55 °C for 30 s, and extension for 32 s at 72 °C, with a final extension at 72 °C for 5 min. PCR amplicons were sequenced bidirectionally by Macrogen Spain (Madrid, Spain) and validated using CHROMASPRO v. 1.32 software (Technelysium, Southport, Queensland, Australia). All obtained sequences were searched through the GenBank database (BLASTn) and the best matches were recorded. PCR and sequencing were considered to have failed after four attempts. All sequences were deposited in GenBank (Table S2).

### 4.3. Integrated Taxonomic Identification Method

**Figure 4:**
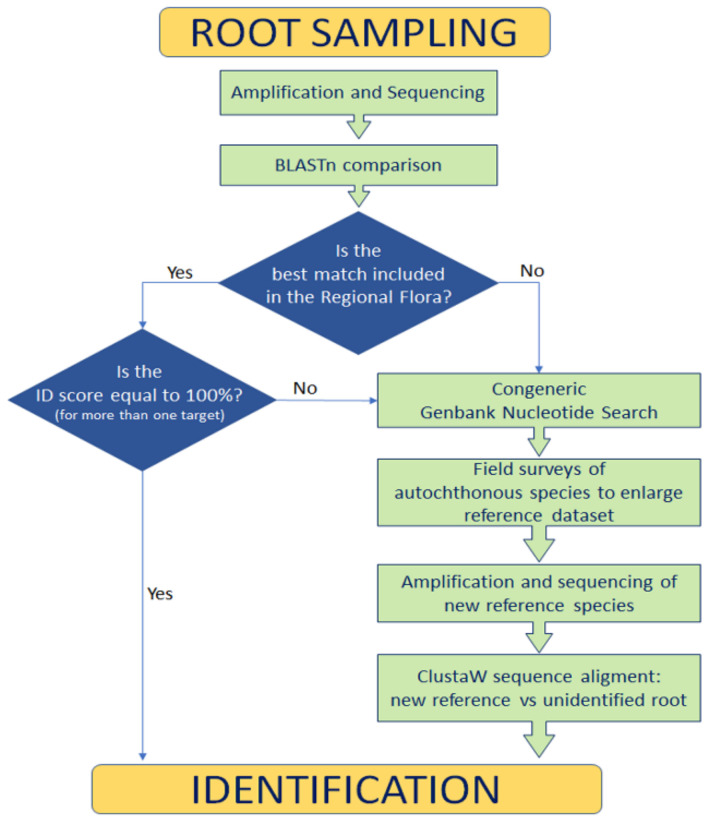
Root identification workflow.

BLASTn best results (Table 2) were cross-referenced with our floristic data of the site and the checklist of the Italian flora [75]. In this way, it was possible to remove some matches at species level corresponding to plants not present in the local flora. To assess the matching reliability, we performed a search in the NCBI nucleotide database for all the congeneric species present in the Latium flora (https://www.ncbi.nlm.nih.gov/nucleotide/, accessed on 6 January 2021). For each congeneric species, we recorded the number of sequences found for each used DNA marker. Because the length of sequences could influence the best score, we reported the minimum and maximum sequence length. Due to the scarcity of genetic information when a plant was represented in the Latium flora as subspecies only, we also included the relative species, and plant data were recorded accordingly (Table S2). To implement the reference database, in summer 2020, after the COVID-19 lockdown and restrictions, we performed a recognition in the field (limited to the visitors’ path) looking for congeneric species. Six plant species were collected and identified according to the analytical keys in [63,76]; their leaves were processed for molecular purposes as previously described for root samples. The obtained sequences were used as additional reference material (Table S4). ClustalW was used to align/compare sequences of reference specimens with unidentified roots. The procedure of the identification workflow is resumed in Figure 4.

## 5. Conclusions

The collected data provide the first assessment of the efficiency of the DNA barcoding approach in the identification of plant rootlets for the preservation of cultural heritage. Despite the positive results, we highlighted the need for some improvements in the GenBank dataset and the selection of specific markers. The collected data also contribute to enhancing the role of herbaceous plant as risk factors for the conservation of hypogeal structures, in specific conditions of high rock porosity and xeric environmental conditions. Further studies are needed to assess species, depth, and risk frequencies, possibly leading, in the near future, to the design of customized control measures.

## Figures and Tables

**Table 1:** Best BLASTn match results obtained for the 14 root samples processed. On the left, the tombs from which the samples were taken. For each sample and target gene, the following are reported in order: blast matches, percentage of identity (%), and accession number as found in GenBank. The most likely identifications are in bold. The order with which species names are reported within cells also considers parameters not shown, such as query coverage, alignment scores, and E value.

Tomb	Sample ID	ITS			*matK*			*rbcL*			*psbA-trnH*		
		BLASTn Match	%	Accession Nr.	BLASTn Match	%	Accession Nr.	BLASTn Match	%	Accession Nr.	BLASTn Match	%	Accession Nr.
**Hunting and Fishing**	H2	*Foeniculum vulgare* *Anethum foeniculoides* *Ridolfia segetum* *Anethum graveolens*	99.84 99.4 98.84 97.34	EU796894 HE602455 GQ148796 GQ148794	*Foeniculum vulgar* *Anethum graveolens* *Ridolfia segetum* *Cuminum cyminum*	100 99.71 99.71 99.57	MK435626 MN216674 HM850713 MG946962	*Apium graveolens* *Prangos trifida* *Foeniculum vulgare* *Anethum graveolens*	99.76 99.76 99.76 99.76	NC_041087 NC_037852 KR011054 MN216674	*Foeniculum vulgare* *Anethum foeniculoides* *Ammi majus* *Petroselium crispum*	99.63 99.63 98.15 98.15	HE659550 MG947083 KU530039 HM596073
	CP01	*Foeniculum vulgare* *Anethum graveolens* *Anethum foeniculoides* *Ridolfia segetum*	99.54 97.24 95.50 98.84	FJ980395 MN257763 HE602455 CG148796	*Foeniculum vulgare* *Anethum graveolens* *Cuminum cyminum* *Apium graveolens*	99.75 99.37 99.12 98.74	JN894477 EU016725 MG946962 AJ429370	*Apium graveolens* *Prangos trifida* *Foeniculum vulgare* *Ligusticum jeholense*	99.81 99.81 99.81 99.63	NC_041087 NC_037852 LT576823 MN652885	*Foeniculum vulgare* *Anethum foeniculoides* *Ammi majus* *Petroselium crispum*	99.63 99.63 98.15 98.15	HE659550 MG947083 KU530039 HM596073
	CP02	*Foeniculum vulgare* *F. vulgare subsp. vulgare* *Anethum foeniculoides* *Ridolfia segetum*	99.18 99.36 98.99 98.34	EU796894 MH645764 HE602455 GQ148796	*Foeniculum vulgare* *Anethum graveolens* *Cuminum cyminum* *Apium graveolens*	100 99.54 99.19 99.08	MG946964 KR011055 MG946962 AJ429370	*Apium graveolens* *Prangos trifida* *Foeniculum vulgare* *Ligusticum jeholense*	99.46 99.46 99.46 99.28	NC_041087 NC_037852 LT576823 MN652885	*Foeniculum vulgare* *Anethum foeniculoides* *Ammi majus* *Petroselium crispum*	99.63 99.63 98.15 98.15	HE659550 MG947083 KU530039 HM596073
**Lotus Flower**	C2	*Centaurea aspera* *Centaurea napifolia* *Centaurea involucrata* *Centaurea pullata*	99.54 98.63 96.35 96.12	DQ319086 DQ319135 DQ319123 DQ319154	*Centaurea diffusa* *Carthamus tinctorius* *Carthamus oxyacantha* *Centaurea nigra*	99.76 99.63 99.63 99.75	KJ690264 HM989751 MG946998 EU385332	*Carthamus oxyacantha* *Carthamus tinctorius* *Centaurea involucrata* *Centaurea melitensis*	99.44 99.44 99.44 99.44	MG946886 KX822074 KC589820 KC589820	*Centaurea aspera subsp.pseudoaerocephala* *Centaurea jacea* *Centaurea bracteata* *Cirsium vulgare*	100 99.13 98.47 98.26	DQ846283 HE966554 FR865076 KY562585
	C1	*Reseda lutea* *R. lutea subsp. lutea* *Reseda crystallina* *Reseda lanceolata*	99.38 99.38 98.55 96.69	KR936125 DQ987095 DQ987088 DQ987099	*Reseda crystallina* *Reseda lutea* *Ochradenus baccatus* *Caylusea hexagyna*	100 99.73 98.64 97.68	FJ212200 FM179932 MT948189 FJ212207	*Oligomeris linifolia* *Ochradenus arabicus* *Reseda lutea* *Reseda crystallina*	99.61 99.61 100 100	MH185895 KX015754 KF724303 FJ212212	*R. lutea subsp. lutea* *Ochradenus baccatus* *Caylusa hexagena*	99.69 90.99 87.21	HE966773 MT948189 MT948187
	LT01	*Centaurea aspera* *Centaurea napifolia* *Centaurea involucrata* *Centaurea pullata*	99.53 98.11 95.74 95.27	DQ319086 DQ319135 DQ319123 DQ319154	*Centaurea diffusa* *Centaurea nigra* *Centaurea calcitrapa* *Centaurea scabiosa*	99.76 99.75 99.75 99.75	KJ690264 JN895178 MK925659 KT249946	*Carthamus* *oxyacantha* *Carthamus tinctorius* *Centaurea involucrata* *Centaurea melitensis*	99.67 99.67 99.67 99.67	MG946886 KX822074 KC589820 KC589820	*Centaurea aspera subsp.pseudoaerocephala* *Centaurea jacea* *Centaurea bracteata* *Cirsium vulgare*	100 99.13 98.48 98.28	DQ846283 HE966554 FR865076 KY562585
	LT3	*Centaurea aspera* *Centaurea napifolia* *Centaurea involucrata* *Centaurea pullata*	99.37 97.95 95.58 95.10	DQ319086 DQ319135 DQ319123 DQ319154	*Centaurea diffusa* *Carthamus tinctorius* *Carthamus oxyacantha* *Centaurea nigra*	99.76 99.63 99.63 99.75	KJ690264 MG946998 KX822074 JN895178	C. *oxyacantha* *Carthamus tinctorius* *Centaurea involucrata* *Centaurea melitensis*	99.70 99.70 99.70 99.70	MG946886 KX822074 KC589820 KC589820	*Centaurea aspera subsp.pseudoaerocephala* *Centaurea jacea* *Centaurea bracteata* *Cirsium vulgare*	100 99.13 98.47 98.26	DQ846283 HE966554 FR865076 MN275426
**Moretti**	M1	*Reseda lutea* *R. lutea subsp. lutea* *Reseda crystallina* *Reseda lanceolata*	99.36 99.38 98.52 95.89	KR936125 DQ987095 DQ987088 DQ987099	*Reseda crystallina* *Reseda lutea* *Ochradenus baccatus* *Caylusea hexagyna*	100 99.73 98.64 97.68	FJ212200 FM179932 MT948189 FJ212207	*Oligomeris linifolia* *Ochradenus arabicus* *Reseda lutea* *Reseda crystallina*	99.42 99.42 99.80 99.80	MH185895 KX015754 KF724303 FJ212212	*Reseda lutea subsp. lutea* *Ochradenus baccatus* *Caylusea hexagena*	100 91.28 87.46	HE966773 MT948189 MT948187
	M2	*Echium italicum* *E. italicum subsp. italicum.* *Echium glomeratum* *Echium asperrimum*	99.48 99.18 99.01 98.84	LC426085 MK321757 MK311754 MK321749	*Echium italicum* *Echium vulgare* *Echium plantagineum* *Echium angustifolium*	99.72 99.44 99.44 99.15	EU599699 MK520026 HM850866 EU599695	*E. vulgare subsp vulgare* *Echium italicum* *Echium plantagineum* *Moltkiopsis ciliata*	100 100 99.76 99.76	HE963457 EU599874 MN157267 KX282888	*Echium italicum* *Echium plantagineum* *Echium vulgare*	98.58 98.53 98.28	LC426222 MG598304 FJ8273162
**Old man**	D4	*Sinapis alba* *Eruca vesicaria* *Rytidocarpus moricandioides*	99.53 92.72 93.49	FJ609733 LC090005 MF192787	SEQUENCING FAIL			SEQUENCING FAIL			*Sinapis alba* *Sinapis arvensis* *Trachystoma ballii* *Erucastrum cardaminoides*	99.46 90.96 90.91 94.00	NC045948 KU050690 AB669922 KJ685143
**Sculptures**	F1	*Ascom. CCFEE 6623* *Ascom. CCFEE 6662* *Plectospherella sp.* *Acremonium nepalense* *Ascom. CCFEE 6624*	99.29 98.10 98.10 97.87 97.87	MT472274 MT472276 KY670795 HG008742 MT472275	PCR FAIL			*Brassica juncea* *Brassica nigra* *Brassica carinata* *Raphanus sativus* *Brassica oleracea*	99.67 99.67 99.67 99.35 99.35	MG872827 AP012989 JF920287 MN056359 LR031888	SEQUENCING FAIL		
	F3	SEQUENCING FAIL			*Verbascum thapsus* *Verbascum carmanicum* *Verbascum kermanense* *Verbascum gabrieliae*	99.29 99.19 99.19 99.19	JN893995 MH885332 MH885331 MH885333	*Verbascum thapsus* *Verbascum thapsus* *Buddleja colvilei* *Phryma leptostachya*	99.82 100 99.08 99.08	KT178130 KJ841648 NC_042766 NC_042727	*Verbascum sinuatum* *Verbascum virgatum* *Verbascum chinense* *Verbascum thapsus*	100 99.73 98.50 98.48	HE699541 KR361652 MT610040 MF348529
**Bartoccini**	02	*Echium italicum* *E. italicum subsp. italicum* *Echium glomeratum* *Echium asperrimum*	99.34 99.17 99.17 99.00	MK321757 LC426085 MK321754 MK321749	*Echium italicum* *Echium vulgare* *Echium plantagineum* *Echium angustifolium*	99.63 99.45 99.45 99.26	EU599699 MK520026 HM850866 EU599695	*Echium vulgare* *Echium plantagineum* *Lobostemon fruticosus* *Echiostachic incanus*	99.53 99.69 99.54 99.54	KF158087 HM849964 AM234929 AM234927	*Echium italicum* *Echium plantagineum* *Echium vulgare*	98.58 98.53 98.28	LC426222 MG598304 FJ8273162
**5512**	01	*Angelica cartilagino-marginata* *Peucedanum japonicum* *Seseli tortuosum* *Ledebouriella seseloides*	97.36 96.93 99.83 96.47	AY548222 KX757777 MG697155 KX757775	*Saposhnikovia divaricata* *Ledebouriella seseloides* *Peucedanum japonicum* *Seseli montanum*	99.66 99.66 99.32 99.43	MK435638 MN539269 KU866531 KM035851	*Ligusticum thomsonii* *Seseli montanum* *Peucedanum praeruptorum* *Saposhnikovia divaricata*	100 100 99.81 99.81	MT409619 KM035851 MN016968 MN539269	*Paucedanum terebinthaceum* *Paucedanum praeruptorum* *Paucedanum ampliatum* *Angelica polumorpha*	94.35 93.95 95.19 93.38	MT671397 MN016968 JN046213 NC041580

**Table 2:** Primer pairs and annealing temperatures used.

Locus	Primer Name	F/R	Sequences 5′-3′	Annealing Temperature	References
*matK*	matK2.1a	F	ATCCATCTGGAAATCTTAGTTC	50 °C	[69]
	matK-3FKIM-r	R	CGTACAGTACTTTTGTGTTTACGAG		
	XF	F	TAATTTACGATCAATTCATTC	50 °C	[70]
	5R	R	GTTCTAGCACAAGAAAGTCG		
	390F	F	CGATCTATTCATTCAATATTTC	53 °C	[71]
	1326r	R	TCTAGCACACGAAAGTCGAAGT		
*rbcL*	rbcLa-F	F	ATGTCACCACAAACAGAGACTAAAGC	50 °C	[69]
	rbcLr590	R	AGTCCACCGCGTAGACATTCAT		
	1F	F	ATGTCACCACAAACAGAAAC	50 °C	[72]
	724R	R	TCGCATGTACCTGCAGTAGC		
*psbA-trnH*	psbA	F	GTTATGCATGAACGTAATGCTC	53 °C	[73]
	trnH	R	CGCGCATGGTGGATTCACAATCC		
ITS	ITS5	F	GGAAGTAAAAGTCGTAACAAGG	55 °C	[74]
	ITS3	F	GCATCGATGAAGAACGCAGC		
	ITS4	R	TCCTCCGCTTATTGATATGC		
	ITS2	R	GCTGCGTTCTTCATCGATGC		

F/R, forward or reverse primers.

## Data Availability

All data regarding this research are available. In supplementary material can be found the accession number of the all obtained sequences.

## Supplementary Materials

The following are available online at https://www.mdpi.com/article/10.3390/plants10061138/s1, Table S1: Root sequences accession numbers, Table S2: Representation of congeneric Latium flora in the NCBI GenBank database, Table S3: Reference plant specimen and sequences accession numbers, Table S4: Tombs in study and samples taken.
