# Supplementary material for: Plant DNA Barcode as a Tool for Root Identification in Hypogea: The Case of the Etruscan Tombs of Tarquinia (Central Italy)
Source: Plants (Basel). 2021 Jun 3;10(6):1138. doi: 10.3390/plants10061138 (PMC8228792; doi:10.3390/plants10061138)
Supplement: Supplementary file 1 [file plants-10-01138-s001.zip › Table S1.pdf]

Table S1. Root sequences accession numbers.

Tomb	Sample ID	ITS	*matK*	*rbcL*	*psbA-trnH*
Hunting and Fishing	H2	MW599329	MW662029	MW662005	MW662047
	CP01	MW599330	MW662030	MW662006	MW662048
	CP02	MW599331	MW662031	MW662007	MW662049
Lotus Flower	C2	MW599332	MW662032	MW662008	MW662050
	C1	MW599332	MW662033	MW662009	MW662051
	LT01	MW599334	MW662034	MW662010	MW662052
	LT3	MW599335	MW662035	MW662011	MW662053
Moretti	M1	MW599336	MW662036	MW662012	MW662054
	M2	MW599337	MW662037	MW662013	MW662055
Old man	D4	MW599338	-	-	MW662056
Sculptures	F1	MW599342	-	MW662004	-
	F3	-	MW662038	MW662014	MW662057
Bartoccini	02	MW599339	MW662039	MW662015	MW662058
5512	01	MW599340	MW662040	MW662016	MW662059
