# Supplementary material for: Plant DNA Barcode as a Tool for Root Identification in Hypogea: The Case of the Etruscan Tombs of Tarquinia (Central Italy)
Source: Plants (Basel). 2021 Jun 3;10(6):1138. doi: 10.3390/plants10061138 (PMC8228792; doi:10.3390/plants10061138)
Supplement: Supplementary file 1 [file plants-10-01138-s001.zip › Table S3.pdf]

Table S3. Reference plant specimen and sequences accession numbers

Species	ITS	*matK*	*rbcLa*	*psbA-trnH*
Centaurea aspera subsp. *aspera*	MW605069	MW662023	MW662017	MW662041
Reseda lutea subsp. *lutea*	MW605070	MW662024	MW662018	MW662042
Diplotaxis tenuifolia	MW605071	MW662025	MW662019	MW662043
Diplotaxis erucoides	MW605072	MW662026	MW662020	MW662044
Verbascum sinuatum	-	MW662027	MW662021	MW662045
Seseli tortuosum subsp. *tortuosum*	MW605073	MW662028	MW662022	MW662046
