# Supplementary material for: Plant DNA Barcode as a Tool for Root Identification in Hypogea: The Case of the Etruscan Tombs of Tarquinia (Central Italy)
Source: Plants (Basel). 2021 Jun 3;10(6):1138. doi: 10.3390/plants10061138 (PMC8228792; doi:10.3390/plants10061138)
Supplement: Supplementary file 1 [file plants-10-01138-s001.zip › Table S4.pdf]

Table S4. Tombs in study and samples taken

Tomb	Coordinates	Sample ID	Place
Hunting and Fishing	42°15′0.2″ N; 11°46′7.3" E	H2	*dromos*, right wall
		CP01	*dromos*, right wall
		CP02	*dromos*, left wall close to entrance
Lotus flower	42°15'1.24" N; 11°46'7.76" E	C2	ceiling, central *columen*
		LT3	right wall
		C1	ceiling, right slope
		LT01	*dromos*
Moretti	42°14'58.6" N; 11°46'13.4" E	M1	ceiling, slope near the entrance, right side
		M2	front wall, right side
Old man	42°14'53.1" N; 11°46'15.2" E	D4	lobby, left corner
Sculptures	42°14'53,04" N; 11°46'15,04" E	F1	*dromos,* right wall
		F3	*dromos,* left wall
Bartoccini	42°14'57,58" N; 11°46'17,20" E	02	entrance, left jamb
5512	42°14'57,36" N; 11°46'14,89" E	01	main chamber
